# HIPPO: HIstogram-based Pseudo-POtential for scoring protein-ssRNA fragment-based docking poses

**DOI:** 10.1186/s12859-024-05733-6

**Published:** 2024-03-26

**Authors:** Anna Kravchenko, Sjoerd Jacob de Vries, Malika Smaïl-Tabbone, Isaure Chauvot de Beauchene

**Affiliations:** Université de Lorraine, CNRS, Inria, LORIA, 54000 Nancy, France

**Keywords:** Scoring function, Protein-ssRNA docking, RRM-ssRNA docking, Fragment-based docking

## Abstract

**Background:**

The RNA-Recognition motif (RRM) is a protein domain that binds single-stranded RNA (ssRNA) and is present in as much as 2% of the human genome. Despite this important role in biology, RRM-ssRNA interactions are very challenging to study on the structural level because of the remarkable flexibility of ssRNA. In the absence of atomic-level experimental data, the only method able to predict the 3D structure of protein-ssRNA complexes with any degree of accuracy is ssRNA’TTRACT, an ssRNA fragment-based docking approach using ATTRACT. However, since ATTRACT parameters are not ssRNA-specific and were determined in 2010, there is substantial opportunity for enhancement.

**Results:**

Here we present HIPPO, a composite RRM-ssRNA scoring potential derived analytically from contact frequencies in near-native versus non-native docking models. HIPPO consists of a consensus of four distinct potentials, each extracted from a distinct reference pool of protein-trinucleotide docking decoys. To score a docking pose with one potential, for each pair of RNA–protein coarse-grained bead types, each contact is awarded or penalised according to the relative frequencies of this contact distance range among the correct and incorrect poses of the reference pool. Validated on a fragment-based docking benchmark of 57 experimentally solved RRM-ssRNA complexes, HIPPO achieved a threefold or higher enrichment for half of the fragments, versus only a quarter with the ATTRACT scoring function. In particular, HIPPO drastically improved the chance of very high enrichment (12-fold or higher), a scenario where the incremental modelling of entire ssRNA chains from fragments becomes viable. However, for the latter result, more research is needed to make it directly practically applicable. Regardless, our approach already improves upon the state of the art in RRM-ssRNA modelling and is in principle extendable to other types of protein-nucleic acid interactions.

**Supplementary Information:**

The online version contains supplementary material available at 10.1186/s12859-024-05733-6.

## Introduction

Protein-RNA complexes play an immensely important role in many cellular processes, including translation, transcription, and post-transcriptional gene expression [1]. The disruption of the binding can lead to tremendous cellular malfunctions [2]. A large part of these protein-RNA interactions involves one of the few conserved RNA-binding domains. In particular, over 50% of all RNA-binding proteins in humans contain an RNA recognition motif (RRM) [3]. This motif is critical for binding to RNA molecules, and to single-stranded RNAs (ssRNA) specifically, making RRM-ssRNA interactions crucial for understanding the underlying mechanisms of various cellular processes.

Although the 3D structure of these complexes provides valuable insights into their functions, the experimental resolution of such structures is a non-trivial task. Computational modelling of the 3D structure of a protein-RNA complex, also known as protein-RNA docking, can facilitate experimental research, by proposing probable 3D structures to be experimentally tested. Many docking methods have been developed specifically for protein-RNA complexes, such as 3dRPC [26], P3DOCK [27] and RnaX [28]. Others have been developed for proteins or small ligands and made compatible with protein-RNA docking, such as AutoDock Vina [29, 30], GRAMM [31], NPDock [32], ZDOCK [33], HADDOCK [34], HDOCK [35]. Unfortunately, protein-ssRNA docking is a more challenging task. The classical docking approaches [4] require an unbound structure as a starting point, but no such structure is available for ssRNA due to its disorder in the unbound state. Therefore, while some RNA–protein scoring functions have been tested on benchmarks that comprise few complexes with ssRNA [36–40], they used bound docking on the RNA side for those cases, which is not doable in a real ssRNA docking case.

On the one hand, to bypass this limitation, one may try to model all possible ssRNA conformations using its sequence, and then dock them. However, ssRNA’s flexibility (~ 8 DOF per nucleotide [5]) makes systematic modelling of ssRNA conformations extremely demanding computationally and borderline impossible for long chains. On the other hand, in recent years, various powerful deep learning techniques ([6–8]) brought breakthroughs to protein–protein [9] and protein–ligand [10, 11] docking. However, deep learning approaches are more challenging to apply to protein-RNA docking, not only due to the relatively low number of solved structures (about 1.16 × 10^4^ protein-RNA structures compared to about 1.776 × 105^5^ protein chains) but also because among all atomic contacts within each structure, the interaction between RNA and protein represents only a tiny fraction. This is even more true for ssRNA, which is only a small subset of RNA, and whose binding modes to proteins have some particularities compared to double-stranded (ds) RNA [12]. A deep-learning-based method RoseTTAFoldNA was recently developed for RNA–protein docking [41]. Its results on 814 cases show a high dependency on the presence of homologous complexes in the training set. On the 7 cases comprising mostly ssRNA at the protein-RNA interface (4PMW, 6YYM, 7A9W, 7A9X, 7M5O, 7B0F, 7OM3), only one case (4PMW) had a model with iRMSD < 4 Å (CAPRI criteria for a correct model [42]), and that case had a close homologue (E-value < 10^−12^) in the training set. The authors acknowledge that small single-stranded nucleic acids are one of the most common causes of poor predictions.

Fragment-based docking handles ssRNA flexibility by subdividing its sequence into fragments that are small enough for their conformations to be exhaustively (including close-to-bound conformation) sampled within a given accuracy threshold. The docking procedure consists of sampling and scoring. Sampling refers to the generation of docking *poses—*certain positions and orientations of particular conformations of the fragment with respect to the protein. A pool of docking poses is sampled for each fragment independently. Scoring is the evaluation of the probability of each pose to be near-native, i.e. correct, followed by ranking. Finally, the presumably best poses of adjacent fragments are assembled into complete structures called docking *models*. In a test case, when the native structure of a complex is experimentally determined, both docking poses and models can be assessed based on their similarity to the corresponding parts of a native structure, and this similarity can be quantified by their ligand root mean squared deviation (LRMSD). The distinction is made between near-native, non-native (incorrect), and intermediate poses/models based on LRMSD thresholds.

For successful docking of the whole RNA chain, at least one near-native pose must be sampled and retained for assembly for each of the fragments. Otherwise, the docking for a given complex will certainly fail at the assembly step. Therefore, fragment-based docking can face two main problems. First, the sampling problem arises when not a single near-native pose is generated during the docking run. Second, the scoring problem arises when none of the sampled near-natives is selected in the list of top-ranked poses. In this case, more poses per fragment must be retained to have a good chance to keep a near-native, which quickly becomes very expensive computationally in the assembly step. In turn, as there are more docking models, identification of the near-native model also becomes more challenging.

There are four existing fragment-based approaches for protein-ssRNA docking (Table 1): RNA-LIM, FBDRNA, RNP-denovo, and ssRNA’TTRACT. RNA-LIM represents each nucleotide by one non-oriented bead and could only predict their position at 15 Å resolution for one example [14]. FBDRNA uses mononucleotide fragments in all-atom representation, docked with MCSS on a pre-defined binding site. While showing discriminative power on nucleotides’ positions, it could not provide accurate models for full oligonucleotides [15]. RNP-denovo, a Rosetta method to simultaneously fold-and-dock RNA to a protein surface, uses the exact position of a few nucleotides [16], which would be unavailable for real-life docking cases. On the other hand, ssRNA’TTRACT, the state of the art, is the most accurate approach that uses only a protein structure and the RNA sequence as input. It uses trinucleotides as RNA fragments and an overlapping criterion based on LRMSD for assembly. Furthermore, when information about conserved protein-RNA contacts are available, ssRNA’TTRACT employs an anchored docking strategy to build the RNA chain incrementally by docking one fragment with contact restraints and using each of its top-ranked poses as an anchor to superimpose subsequent fragments [17]. This strategy tackles the sampling problem for the fragments. ssRNA’TTRACT uses the ATTRACT docking engine and a library of RNA trinucleotide conformations developed in our research group [18, 19]. A coarse-grained force field with Lennard–Jones type energy function with soft potential [20] is used for both sampling and scoring. In the coarse-grained representation, the RNA fragments and the protein are represented as sets of pseudo-atoms, called *beads*, each of which stands for a small group of real atoms.

**Table 1:** Comparison of existing ssRNA-protein fragment-based docking methods

Method	Advantages	Disadvantages
RNA-LIM	Simplicity (single nucleotide representation)	Source code not available for download
		Can predict only global binding site
		No orientation of nucleotides
FBDRNA	Simplicity (single nucleotide representation)	Source code not available for download
	Created for design	Poor docking predictions (no success on chains longer than 3 nucleotides)
	Modified nucleotides accepted	
RNP-denovo	Protocol available for download	Requires as input the precise coordinates of at least the first and the last nucleotides
	Gives acceptable RMSDs in top-100 docking poses (fold and dock)	
ssRNA'ATTRACT	Protocol available for download	Works at the fragment level
	Gives acceptable RMSDs while performing completely blind docking	Requires a lot of docking poses
		Produces many models
ssRNA'ATTRACT + HIPPO	Protocol available for download	Works at the fragment level
	Gives acceptable RMSDs while performing completely blind docking	Requires a lot of docking poses
	Provides enrichment of good models compared to ssRNA'ATTRACT alone	

Despite its capabilities, ssRNA’TTRACT is still constrained by the aforementioned limitations. As the current ATTRACT protein-RNA scoring function was not designed to tackle ssRNAs specifically and its parameters were optimised back in 2010 on dsRNA alone, there is considerable potential for enhancement. Here we present HIstogram-based Pseudo-POtential (HIPPO), which aims to distinguish between near-native and non-native protein-ssRNA docking poses. HIPPO was derived from a fragment-based docking benchmark of 57 experimentally solved RRM-ssRNA complexes, corresponding to 217 overlapping ssRNA trinucleotide fragments in complex with an RRM. Using cross-validation, HIPPO achieved a threefold enrichment (60% of all near-native poses in the 20% top-ranked poses) for 53% of the fragments, versus only 26% with the current state-of-the-art ATTRACT scoring function (ASF).

We tested HIPPO on two sets of complexes outside of the initial benchmark used for HIPPO’s development. We also tested two of the state-of-the-art RNA–protein scoring methods—DARS-RNP [36] and DRPScore [39] (chosen for their availability) on our docking poses and compared the results with those of the ATTRACT scoring function and HIPPO. Lastly, we tested two popular free docking methods—3dRPC [26] and AutoDock Vina [29, 30], and compared these results with those provided by the ATTRACT docking engine.

The source code of HIPPO is available via https://github.com/AnnaKravchenko/hippo. This repository contains the HIPPO scoring parameter set along with scripts to score protein-RNA docking models, together with an application guide; the list of used data can be found in Additional file 1: Table S2.

## System and methods

**Fig. 1:**
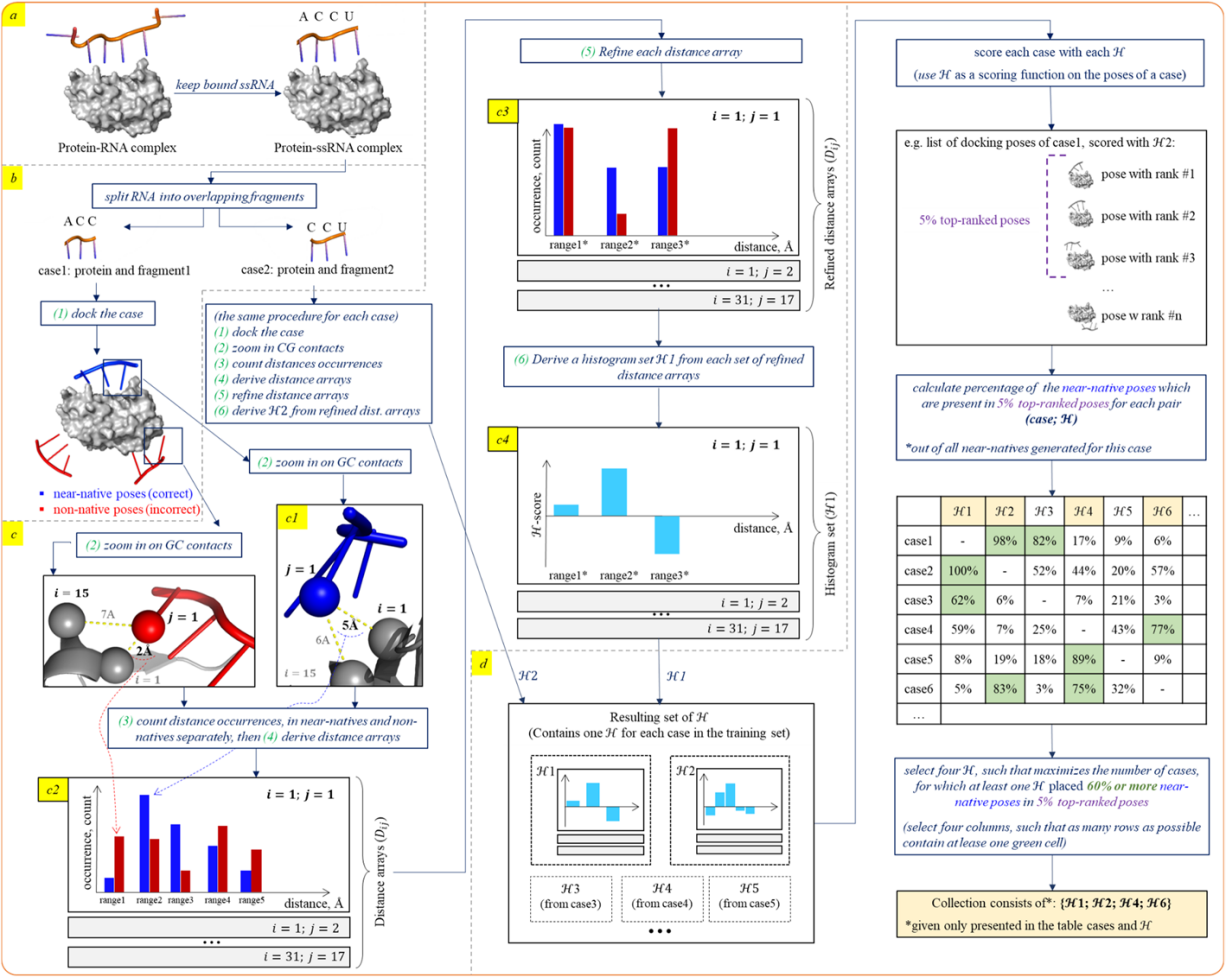
Graphical pipeline for building HIPPO as a collection of four histogram sets (${\mathcal{H}}$). **a **Transition from a protein-RNA complex to a protein-ssRNA complex with ssRNA that is at least 3 protein-bound nucleotides long. This step was achieved using ProtNAff. **b** Creation of a pool of labelled docking pose using ATTRACT. Each protein-fragment case of protein-ssRNA complex is docked and each docking pose is labelled as near-native or non-native. **c** Construction of the distance arrays, refinement of the distance arrays and derivation of the histogram set ${\mathcal{H}}$ from refined distance arrays. The frequency of occurrences of individual bead-bead distances within a single pool of docking poses are captured within distance arrays, one array per each pair of bead types. **c1** Close-up of contacts between RNA bead *j* = 1and protein bead *i* = 1 and *j* = 15. **c2** An intuitive schema of the distance array for the pair of bead types (*i* = 1; *j* = 1) is shown as an expanded plot. The distance ranges are shown on the x-axis, and the numbers of occurrences of the distances are shown on the y-axis. For each distance range, the number of occurrences for the near-native poses is displayed as a blue bar, and for the non-natives as a red bar. The blue dashed line from c1 to c2 shows the contribution of the contact to the near-native distance array, range2. The other distance arrays (for other pairs of bead types) are not shown (collapsed). **c3** An intuitive schema of the refined distance array for the pair of bead types (*i* = 1; *j* = 1) is shown as an expanded plot. Due to the relatively low number of near-native contacts in *range*1, it is merged with *range3*, forming a new *range*1^*^. The following *range*3, which contains a sufficient number of near-native contacts, remains unchanged and is renamed as rang*e*2^*^ to preserve the range order. Finally, *range*4, which also contains an insufficient number of near-native contacts, is merged with *range5* to form a new *range*3,*. **c4** An intuitive schema of the histogram set ${\mathcal{H}}$, derived from the refined distances arrays, which are in turn built from the pool of the docking poses of the case1. A histogram for the pair of bead types (*i* = 1; *j* = 1) is shown as an expanded plot, other histograms are collapsed. **d** Schematic pipeline of the partitioning algorithm, employed to derive a collection of four histogram sets out of all sets

Here we first present the dataset that was built and used to the train and validate HIPPO. Next, we present step-by-step the process of constructing a set of scoring parameters in the form of a histogram set ${\mathcal{H}}$ and the process of building the final collection of several ${\mathcal{H}}$ (Fig. 1). HIPPO is based on the hypothesis that there exists a collection of scoring parameter sets (as opposed to a single parameter set) that can be used to effectively rank near-native protein-ssRNA docking solutions. HIPPO’s parameters are derived analytically from contact frequencies in near-native versus non-native docking poses. These contact frequencies, derived from four different sets of docking poses, are discretised by a particular set of cutoffs into histograms, leading to a collection of four histogram sets ${\mathcal{H}}$ that together form the HIPPO scoring potential. Thus, HIPPO is a composite protein-ssRNA scoring potential: typically, the top5% of the poses according to each histogram set are combined, selecting 20% of all docking poses in total. To streamline the process from dataset construction to the generation of final scoring parameters, we decided to focus exclusively on the RRMs, as this domain of the protein is particularly important for studying protein-ssRNA interactions and is present in many (approximately 65%) of the available protein-ssRNA structures. This allows us to provide proof of principle that the scoring function can indeed be improved using our method. However, the developed method and protocol can be applied to a wider benchmark, and more importantly, to other types of protein-nucleic acid interactions in the future.

### Data

#### RRM-ssRNA benchmark

The number of experimentally solved protein-ssRNA structures is considerably low compared to protein–protein structures. We gathered all available data and built an up-to-date benchmark of experimental 3D structures of RRM-ssRNA complexes from the Protein Data Bank (PDB) by (i) downloading all experimentally solved (either NMR or X-RAY with resolution 3 Å or higher) protein-RNA complexes and (ii) applying ProtNAff [18] in order to retrieve complexes with 3 or more consecutive protein-bound single-stranded nucleotides (Fig. 1a). We considered a nucleotide to be protein-bound if at least 5 pairs of RRM-RNA heavy atoms were located within 6 Å from each other. Lastly, we filtered out complexes whose protein does not contain any RRM domain, according to the InteR3M database [21]. The resulting benchmark consists of 81 RRM-ssRNA complexes, released before February 2021.

#### Dataset of docking poses

**Fig. 2:**
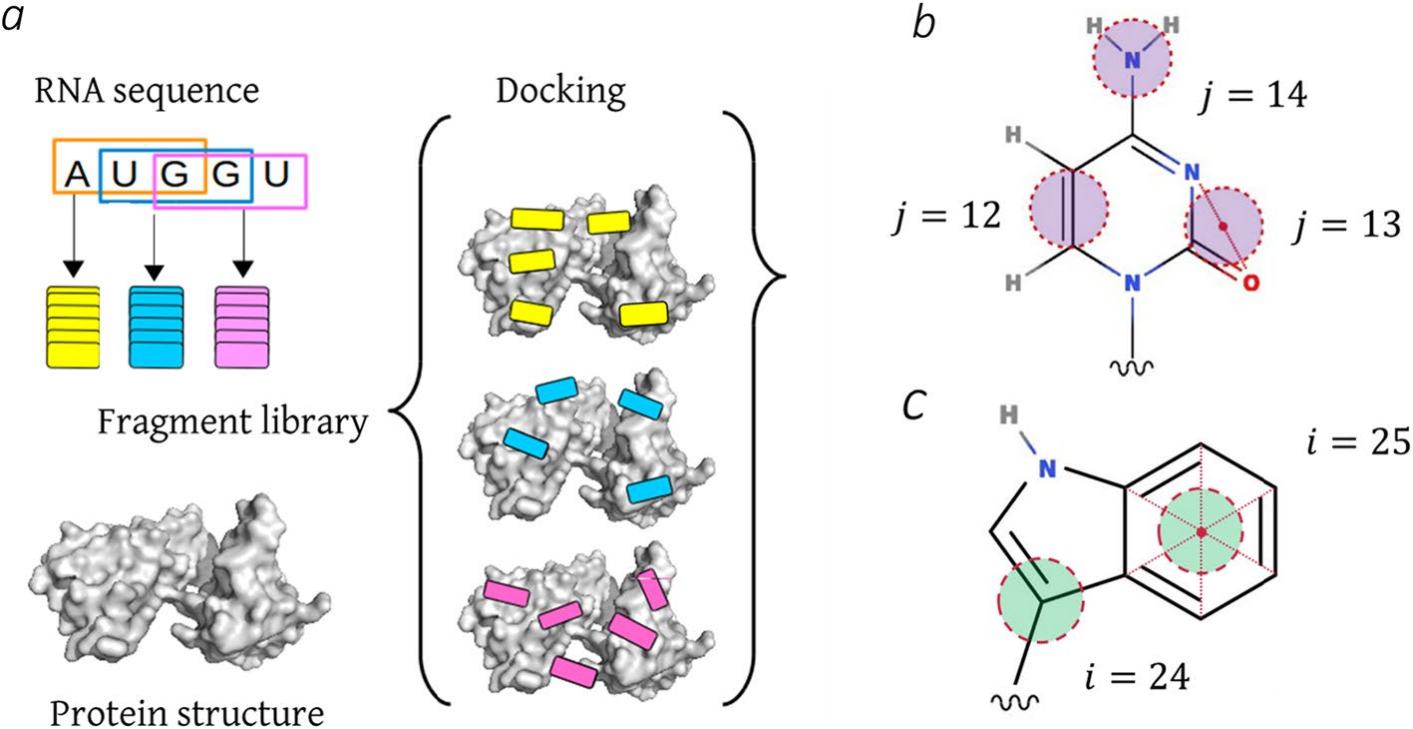
a Schematic image of the fragment-based docking protocol [23], carried out by ATTRACT docking engine; b schematic image of the Cytosine nucleobase in coarse-grained representation. RNA beads are shown in purple, alongside their indexes [20]; c schematic image of the Tryptophan side chain in coarse-grained representation. Protein beads are shown in green, alongside their indexes [20]

From the benchmark, we created a dataset of labelled docking poses (Fig. 1b). We used the ATTRACT docking engine and library of RNA trinucleotide conformations [22] to dock each entry (each RRM-ssRNA complex) of the benchmark, by docking each overlapping trinucleotide fragment (e.g. chain AUCG =  > fragment AUC and fragment UCG), following the procedure described in [23] (Fig. 2a). For each fragment, a randomly selected conformation from the trinucleotide library was placed at each of 3 × 10^7^ predefined starting points located within 30 Å from the center of mass of the bound and rigid protein, with a random 3D rotation. Then the position of each starting pose was minimised using gradient descent. Redundant poses (RMSD < 0.2 Å) were filtered out of the resulting pool before scoring. The remaining docking poses were scored, and the 10^7^ top-ranked poses were retained. Each pose was labelled as near-native if its LRMSD was under 5 Å; as non-native if its LRMSD was over 7 Å; as intermediate otherwise. The duration of the docking procedure depends on the size of the protein, e.g. on 30 CPUs, docking takes ~ 7 h for a protein comprising ~ 1500 atoms, and ~ 12 h for a protein comprising ~ 2500 atoms.

We used such relatively soft thresholds to lower the number of cases for which the sampling problem (zero near-native poses sampled) has arisen. For example, the more strict thresholds [3 Å;5 Å] resulted in 41% of cases with the sampling problem, versus just 8% with [5 Å; 7 Å]. To minimise the noise in the dataset, 60/479 cases (12.5%) where the number of sampled near-natives was less than 100 were excluded. This led to a set of 419 RRM-trinucleotide fragment docking cases. Note that in the case of multiple fragments with the same sequence bound to the same RRM, only a single docking is necessary.

#### Coarse-grained representation

As mentioned before, in the coarse-grained representation, groups of atoms are represented by beads. In the used representation, 31 bead types are used to represent proteins (2 for backbone and 0–2 for side chain) and 17 bead types are used to represent RNA (1 for phosphate group, 2 for sugar and 3–4 for base), leading to a maximum of 527 pairs of bead types [20]. Protein beads are denoted by index *i* and RNA beads are denoted by index *j* (Fig. 2b, c).

#### Redundancy

In order to eliminate possible dataset bias, we performed a redundancy check at the contact level, by comparing *i*-bead to *j*-bead distances within 6 Å in the native poses of the protein-fragment cases. If such distance sets were very similar for two cases, these cases were considered redundant, and one of them was removed from the dataset. The final dataset consists of 217 RRM-fragment cases, with 10^7^ labelled docking poses per case. Its corresponding benchmark consists of 57 RRM-ssRNA complexes and can be found in Additional file 1: Table S1.

#### Training and test sets

We separated the dataset into pairs of training and test sets based on protein sequence similarity, in a leave-homology-out procedure. Our sequence similarity threshold was 40%. We selected a random protein-ssRNA complex from the benchmark along with all other complexes whose protein sequence similarity was greater than 40%. All data cases derived from these complexes (protein-fragment structures along with their docking poses) became the test set. The remaining data cases formed the corresponding training set. We repeated this procedure iteratively until each of the benchmark complexes was in one of the test sets. To prohibit repetitive and near-repetitive (training; test) pairs, we ensured that the first randomly selected case in each iteration did not belong to any of the previous test sets. All statistics reported in this paper correspond to the evaluation of HIPPO on the test sets, where for each test set the four histogram sets ${\mathcal{H}}$ derived from the corresponding training set were used. The final collection consists of 29 (training; test) pairs and can be found in Additional file 1: Table S2.

### Creation of histogram set ${\mathbf{\mathcal{H}}}$

The main steps—detailed thereafter—to obtain a scoring histogram set ${\mathcal{H}}$ are as follows:

1. construction of the *distance arrays* containing the number of occurrences of each bead-bead distance, in near-native vs in non-native poses (ignoring intermediate ones), for each pair of bead types (*i*, *j*) independently (Fig. 1c);
2. refinement of the distance arrays to ensure that each of them provides a sufficient signal (Fig. 1c3);
3. derivation of ${\mathcal{H}}$ from the distance arrays, one histogram per distance array (Fig. 1c4).

#### Histogram definition

Let’s denote the bead types representing the protein by index $i \in \left\{ {1,2, \ldots 31} \right\}$, and the bead types representing the RNA by index $j \in \left\{ {1, \ldots 17} \right\}.$. Also, let’s define initial distance ranges by applying discretisations of 0.25 Å and 1.5 Å to the intervals [2 Å; 7 Å] and [7 Å; 14.5 Å] respectively. Such design of distance ranges allows to capture close-range interactions with high precision and to generalise long-range interactions. The resulting set contains 27 ranges: {(0, 2], (2, 2.25], …,(14.5, 999)}.

A distance array *D*_*ij*_ with the dimension 27 × 2 is designed to capture the number of occurrences of all (*i*, *j*) distances within a pool of docking poses. The rows *d*_*k*_, $k = 1 \ldots 27$, of *D*_*ij*_ correspond to the distance ranges. Each element of *D*_*ij*_ contains the count of distances within the indicated range. Elements *d*_*k*1_ in the first column account for the distances in near-native poses only, while elements *d*_*k*2_ from the second column capture distances in non-native poses (Fig. 1c2).

To ensure that in each *D*_*ij*_ there are enough examples coming from near-native poses in each distance range to provide a sufficient signal, we set a threshold w for a minimum number of occurrences in near-natives *d*_*k*1_. The threshold value is empirical and is determined individually for each (*i*; *j*) pair as 1/60 of all distances counted in near-native poses:

$$w_{ij} = \frac{{A_{ij} }}{60},$$

where $$A_{ij} = \sum\nolimits_{k} {d_{k1} } , \forall d_{k1} \in D_{ij}$$.

For each *D*_*ij*_, if $d_{k1} < w_{ij}$, then the rows starting from *k*th and beneath are summed until their sum exceeds the threshold. The new row resulting from the summation replaces the original row. This process is repeated until all values in the first column of the resulting array exceed the threshold. The resulting refined distance array $D_{ij}^{*}$ has dimension qx2, where $q \le 27$, and may vary for different (*i*; *j*) pairs. (Fig. 1c3). Note that for each (*i*; *j*) we must save the resulting set of refined distance ranges for further application of the histogram.

Finally, the following formula, inspired by the logarithm of the odds ratio, is used to obtain individual histograms H_ij_ from the corresponding $D_{ij}^{*}$ (Fig. 1c4):

$${\text{H}}_{{{\text{ij}}}} = \left[ {\ln d_{x1}^{*} - \ln d_{x2}^{*} - \left( {\ln A_{ij} - \ln B_{ij} } \right)} \right],$$

where $$x = 1 \ldots q, \forall x\left[ {d_{x1}^{*} ,d_{x2}^{*} } \right] \in D_{ij}^{*} , B_{ij} = \sum\nolimits_{k} {d_{k2} } , \forall d_{k2} \in D_{ij}$$.

The dimension of *H*_*ij*_ is qx1. We define ${\mathcal{H}}$ as the set of individual histograms *H*_*ij*_ for all (*i*; *j*) pairs, which are present in at least one pose out of the input pool of the docking poses.

Since 10^7^ poses is a rather large pool, poses with vastly different ranks could possess different features. To account for this possibility, we divided the initial pool of poses into 3 sub-pools according to the rank of the poses: [0, 99999], [10^5^, 999999], [10^6^, 10^7^]. Each *D*_*ij*_ and subsequently each *H*_*ij*_ consists of three parts, built on poses from the corresponding rank-based sub-pool.

#### Scoring with ${\mathcal{H}}$ and scoring assessment

To score a pose using ${\mathcal{H}}$, we count the occurrences of distances for each (*i*; *j*) pair within each of the refined ranges, within each rank-based sub-pool. This information is stored in a qx1 array *R*_*ij*_. The histogram-based score of a pose is calculated using the following formula:

1 $$S_{pose} = \mathop \sum \limits_{i} \mathop \sum \limits_{j} R_{ij} \cdot H_{ij}^{T} ,$$

In simpler terms, for every bead-bead distance in a pose that falls in one of the refined ranges, a corresponding sub-score is assigned. This process is repeated for each rank-based sub-pool separately. The sum of all sub-scores is the final histogram-based score of a pose.

To evaluate the performance of ${\mathcal{H}}$ for a data case, we score all docking poses from the pool of 10^7^ poses using formula (1) and rank the poses by their score in a descending order. Then we select the 5% of top-ranked poses and calculate the fraction of all near-native poses that are present in this selection. An ${\mathcal{H}}$ is labelled as successful for a given data case if this value exceeds 60%. Likewise, we can say that a given case is successfully scored by current ${\mathcal{H}}$. Scoring of 10^7^ poses with ${\mathcal{H}}$ takes ~ 11–15 min on 2 CPUs, depending on the number of histograms in ${\mathcal{H}}$.

### Collection of ${\mathbf{\mathcal{H}}}$

**Fig. 3:**
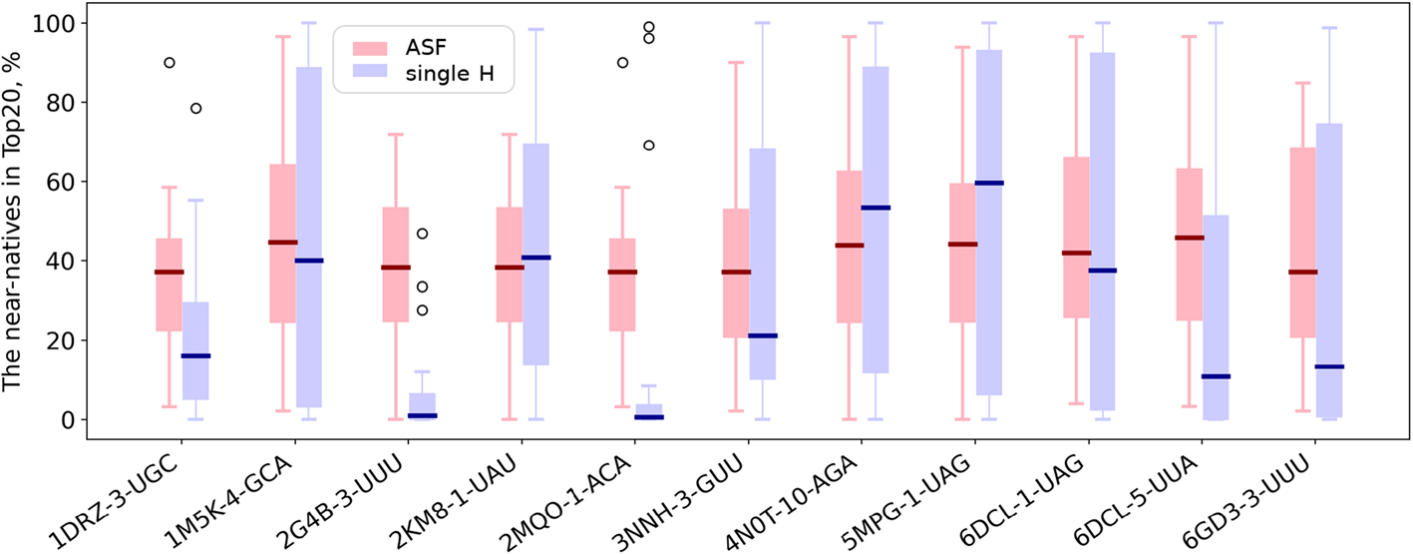
rotein chains) but also because among all atomic contacts Comparison of the percentage of near-natives selected by a single ${\mathcal{H}}$ versus ASF. Each pair of adjacent boxes shows the distribution of the results produced by a corresponding ${\mathcal{H}}$ (purple) and ASF (pink) on the relevant for a given ${\mathcal{H}}$ test set(s) (sets used for the collection to which given ${\mathcal{H}}$ belongs), for a range from 0 to 100% of the near-natives in the 20% top-ranked poses

Initial analysis revealed that a single ${\mathcal{H}}$ was not sufficient to account for the diverse protein-ssRNA binding modes (Fig. 3). Therefore, we opted for the creation of a small collection of ${\mathcal{H}}$, where each ${\mathcal{H}}$ is successful on a subset of the cases. When applied simultaneously, the collection should cover the majority of cases, except for a few outliers. The collection is created by selecting several best-performing ${\mathcal{H}}$, such that maximises the number of successfully scored cases in the training set. The full procedure is detailed in the next section (§2.3.1).

Because in a real-life docking case, there will be no indication of which ${\mathcal{H}}$ from the collection is best suited for scoring, the case must be scored by all ${\mathcal{H}}$ and results must be pooled together (see §2.3.2). As the collection size increases, so does the chance of overfitting. For this reason, we have empirically limited the number of ${\mathcal{H}}$ to four per collection. Changing this number (N) had only limited influence: the success rates for N = 3, N = 4, N = 5 and N = 6 were 40%, 44%, 45% and 37%, respectively. Note that here, the selection of N ${\mathcal{H}}$ was performed simply by maximising the fraction of cases where at least 60% of the near-natives was in the top 20/N % of at least one ${\mathcal{H}}$, and that these success rates correspond to that fraction. In contrast, in the final evaluation (Table 2), duplicate selected poses were eliminated, selecting more poses until 20% was reached, thus increasing the success rate from 44 to 53%.

**Table 2:** Comparison of the performance of HIPPO vs ASF on the 217 cases (29 test sets, 57 complexes)

Comparison criterion	ASF	HIPPO
% of near-natives in TopC/Top20, averaged over all test cases	43	55
Success rate (%) over all cases	26	53
Average highest % of near-natives in TopC/Top20 among the cases of a complex, over all test cases	60	72
Nb of complexes with the > 80% of near-natives in TopC/Top20 for at least one fragment	9	33
Nb of cases with > 80% of near-natives in TopC/Top20	15	75

#### Partitioning algorithm

While deriving a collection of four ${\mathcal{H}}$–${\mathcal{H}}_{1} ,{\mathcal{H}}_{2} ,{\mathcal{H}}_{3}$ and ${\mathcal{H}}_{4}$—we partition the training cases into four subsets, plus a subset of outliers. This procedure is implemented as follows (Fig. 1d):

1. Derive ${\mathcal{H}}$ for each case individually;
2. Score each case with each ${\mathcal{H}}$;
3. For each pair (case; ${\mathcal{H}}$), calculate the percentage of the near-natives that end up in the 5% of top-ranked poses. If the calculated value is over 60%, then label this case as successfully scored by the given ${\mathcal{H}}$;
4. Select the four ${\mathcal{H}}$ that maximise the total number of successfully scored cases. This is the resulting collection.

Now, each training case either is associated with its best-performing ${\mathcal{H}}$ in the resulting collection or ends up in the set of outliers.

#### Scoring with collection and evaluation strategy

**Fig. 4:**
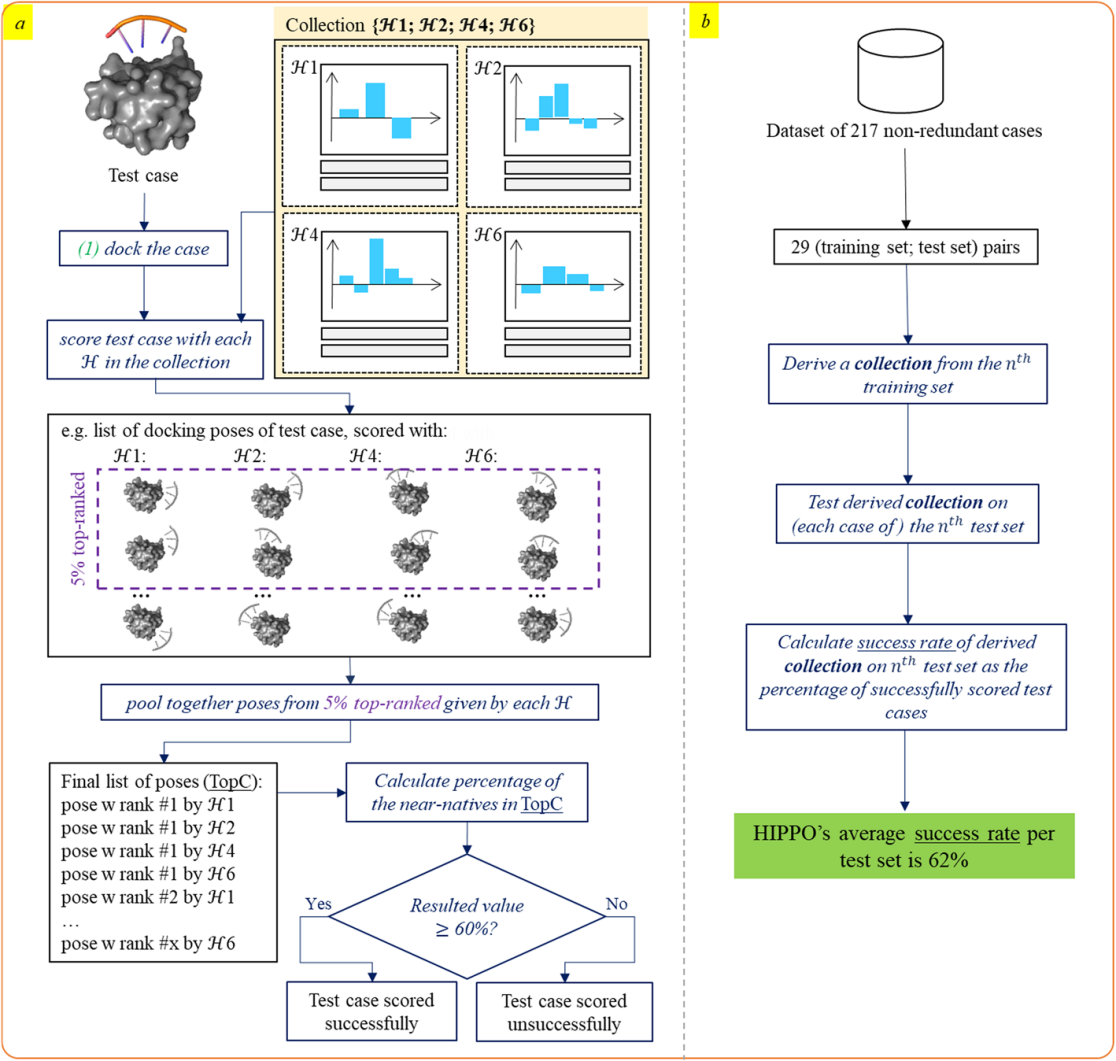
Graphical pipeline for **a** testing a collection on a test case. **b** The complete workflow. The creation of pairs of training and test sets is based on the protein’s sequence similarity: proteins with sequence similarity of 40% or higher are never present in both training and test sets

To score a case with a collection (Fig. 4a), we score its docking poses with ${\mathcal{H}}_{1} ,{\mathcal{H}}_{2}$ and ${\mathcal{H}}_{4}$ separately using (1). Then, for each ${\mathcal{H}}$, around 5% of its top-ranked poses are selected and pooled together in TopC (where “C” stands for a collection). If the same pose is present in several scorings, only its highest rank is kept. The size of the TopC should be equal to 20% of all sampled poses. The resulting set of poses TopC is expected to contain the best ones (the poses outside of TopC are dismissed). As mentioned before, scoring 10^7^ poses with 1 ${\mathcal{H}}$ takes ~ 11 to 15 min on 2 CPUs. Pooling top-ranked poses takes under 1 min on 4 CPUs.

To evaluate the performance of the collection for a case, the fraction of all near-native poses that end up in TopC is calculated. If this value exceeds 60%, then the collection is successful for a given data case.

### Application of HIPPO and BP to new complexes

After assessing the performance of HIPPO in a leave-homology-out procedure, we derived a final version of HIPPO using an entire RRM-ssRNA benchmark, described in §2.1.1. The resulting collection was identical to the one derived from training set 4, comprising potentials derived from the following protein-trinucleotide cases: 1M5K-GCA (protein: C_1_92; trinucleotide: B_38_40), 5MPG-UAG (A_1_97; B_2_4), 4N0T-AGA (A_1_363; B_20_22), 6DCL-UUA (A_1_B_171; C_7_9). For brevity, we refer to these potentials as ${\mathcal{H}}_{1} ,{\mathcal{H}}_{2} ,{\mathcal{H}}_{3}$ and ${\mathcal{H}}_{4}$ onwards. To assess the generalisability of HIPPO, we applied it to:

- A new benchmark of RRM-ssRNA complexes not used during the creation and testing of HIPPO, titled ‘newRRM’;
- A protein-ssRNA benchmark that did not contain any RRM domain, titled ‘nonRRM’.

Alongside HIPPO, we also evaluate the performance of the best-performing potential (BP, described in §3.2.2), which involves the identification of the best-performing potential out of ${\mathcal{H}}_{1} ,{\mathcal{H}}_{2} ,{\mathcal{H}}_{3}$ and ${\mathcal{H}}_{4}$ for each protein-trinucleotide case, currently possible only in a test case.

#### Benchmark of new complexes

Similarly to the initial benchmark for HIPPO, all experimentally solved protein-ssRNA structures in the new benchmarks are solved with NMR or X-RAY with resolution 3 Å or higher and contain a 5-nucleotide or longer ssRNA sub-chain, bound to the protein (i.e. at least 5 pairs of protein-RNA heavy atoms are located within 6 Å from each other). The benchmark ‘newRRM’ consists of 6 RRM-ssRNA complexes that were deposited to PDB after the date the initial HIPPO benchmark was collected (after February 2021) and before December 2023. This set consists of 29 distinct data cases (Additional file 1: Table S5); The benchmark ‘nonRRM’ consists of 150 protein-ssRNA complexes (519 cases) (Additional file 1: Table S6). All proteins in this set do not contain an RRM domain, which was verified using InteR3Mdb [21]. These complexes were deposited to PDB before August 2023. All cases of this subset are non-redundant on the bead-bead contact level, as described in §2.1.4, with each other and with cases used for the training and testing of HIPPO.

#### Protocol for new complexes

All complexes were docked using ATTRACT, with the same setting used for HIPPO derivation, described in §2.1.2. For each protein-trinucleotide case, the 10 million docking poses top-scored by the ASF were retained. Next, the poses were scored and ranked by each of the 4 potentials of HIPPO separately. For the application of HIPPO, approximately the top5% of each ranking were pooled together, removing redundant poses until the total of 20% (2,000,000 poses) was reached. For the application of BP, the top20% given by the BP was taken.

The scoring is considered successful/very successful if the top20% contains at least 60%/80% of all sampled near-native poses (LRMSD < 5 Å).

## Results

In this study, we developed a new protocol for deriving scoring parameters for molecular docking poses, based on distances between RNA and protein beads, in the form of a collection of four histogram sets (${\mathcal{H}}$). We applied it to create HIPPO, a novel scoring function specifically for RRM-ssRNA fragment-based docking. To achieve this goal, we split every available RRM-ssRNA structure into RRM-fragment cases (fragments of 3 consecutive bound nucleotides), for each of which 10^7^ docking poses were generated using the ATTRACT docking engine. Our initial benchmark consisted of 479 fragments from 81 complexes. Out of these, 262 fragments were unusable for training because of a sampling problem (less than 100 near-native poses sampled) or because of redundancy between fragments on the contact level (6 Å), resulting in a dataset of 217 well-sampled non-redundant cases, coming from 57 RRM-ssRNA complexes. Within the resulting dataset, the average number of sampled near-native poses is 9112 and the median is 3145 (out of 10 million). To assess how HIPPO performance would generalise to new data cases (Fig. 4b), we used the leave-homology-out cross-validation strategy: 29 pairs of training and test sets were formed based on RRM sequence similarity. The size of the test set depended on the number of cases derived from each RRM-ssRNA complex of a given RRM and varied from 1 to 33 cases per set.

For a given pair of test and training sets, for each case in the training set, we derived an ${\mathcal{H}}$ by analysing the frequencies of bead-bead distances in the near-native (LRMSD < 5 Å) vs non-native (LRMSD > 7 Å) docking poses, and we applied it to each of the other cases in the training set. We selected the collection of four ${\mathcal{H}}$ sets that maximised the number of training cases for which at least one ${\mathcal{H}}$ ranks 60% of all near-native poses in the 5% top-ranked poses. Then, the collection was applied to the test cases, and the best of the 4 ranks for each pose was retained to obtain the 20% top-ranked poses (TopC). The collection was considered to be successful on a test case if at least 60% of all near-native poses were in TopC. Changing 4 to a different number had only a limited effect on the results (see §2.3).

### General performance

**Fig. 5:**
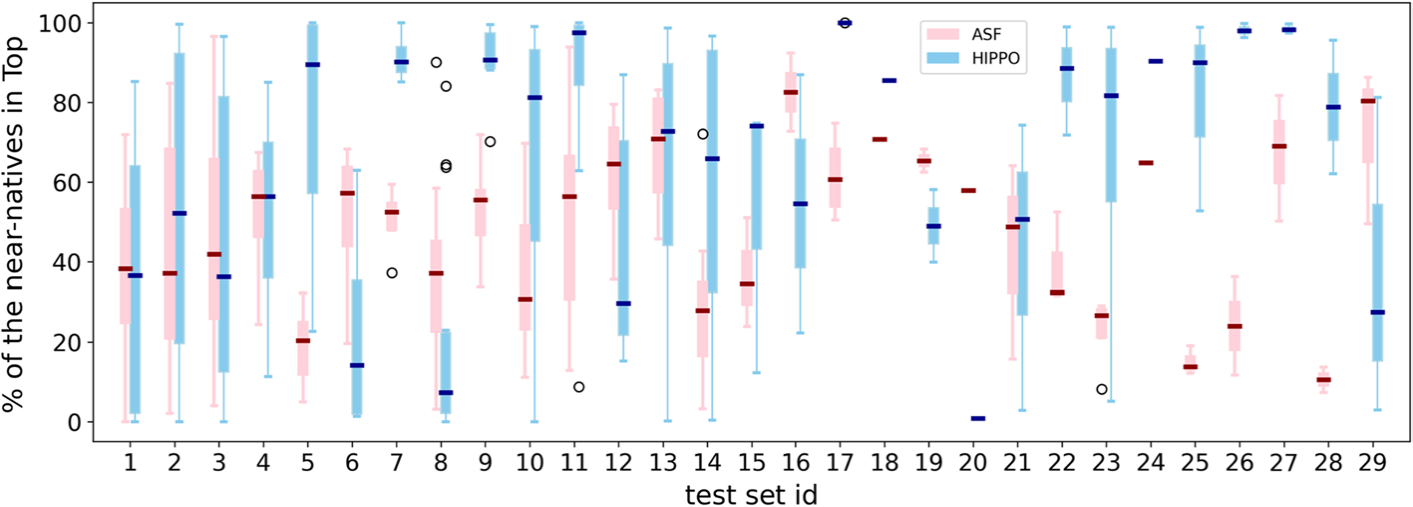
Comparison of the percentage of selected near-natives by collections vs ASF on the test sets. Each pair of adjacent boxes shows the distribution of the results produced by a corresponding collection (blue) and ASF (pink) on one of the 29 test sets, for a range from 0 to 100% of the near-natives in the corresponding Top (TopC/Top20 respectively)

We applied the described protocol to each of the 29 training sets and derived 29 collections of four ${\mathcal{H}}$. We then applied these collections to the cases in the corresponding test sets and compared the percentages of near-natives selected in TopC with HIPPO and in the 20% top-ranked with the ATTRACT scoring function (ASF) (Table 2; Fig. 5). For 19/29 test sets, HIPPO’s median is higher than ASF’s median. At least 60% of all near-natives selected (a threefold enrichment compared to random scoring) for more than half of the RRM-fragment test cases with HIPPO, versus a quarter with ASF (53% vs 26% of the test cases respectively). In one-third of the test cases, we even observed a fourfold enrichment (80% of near-natives selected) with HIPPO, something which is rarely achieved by ASF (38% vs 7% of the test cases respectively).

**Fig. 6:**
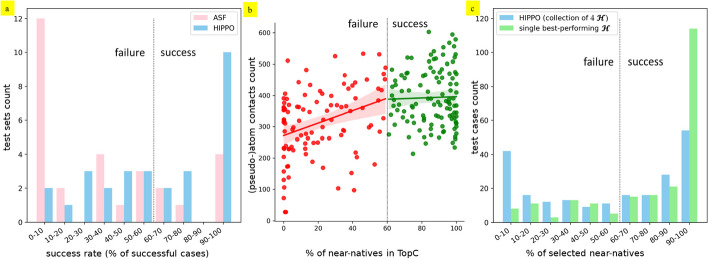
**a** Distribution of the success rate per test set, achieved with ASF (pink) and HIPPO (blue). The black dotted line indicates the threshold of a threefold enrichment compared to random sampling. **b** Relation between the number of contacts in a protein-fragment structure vs the percentage of near-natives in TopC achieved by HIPPO. **c** Distribution per test case of the percentage of near-natives selected by a collection of four ${\mathcal{H}}$ (blue) versus by a single best-performing ${\mathcal{H}}$ (green)

To ensure that our results were not skewed by cases coming from one or a few largest test sets, we compared the average success rates over the test sets and found 62% and 34% respectively (Fig. 6a). Notably, for 10/29 test sets the success rate of HIPPO is very high (from 90 to 100), while ASF demonstrates the lowest possible rate (from 0 to 10) for 12/29 test sets.

#### Best-scored fragment per complex

We found a positive correlation (Pearson correlation, *r* = 0.43, Fig. 6b) between the number of protein-fragment contacts under 5 Å and the percentage of near-natives in TopC, which complies with the cold/hotspot theory. To perform anchored fragment-based docking, at least one fragment per complex must be well-docked. We thus analysed the distribution of successes among the complexes, with HIPPO and ASF. The number of complexes with at least one successfully scored fragment increased from 54% with ASF to 75% with HIPPO. With the success criterion raised to 80% of the near-natives selected (a fourfold enrichment), the compared success rate percentages still increased from 16% with ASF to 58% with HIPPO. Moreover, the enrichment for the best-scored fragment per complex was increased with HIPPO compared to ASF in 68% of complexes. On average, for the best-scored fragment of each complex, HIPPO selects an additional 19% of all near-natives compared to ASF.

### Analysis of the collections

To assess the gains of using a collection (four ${\mathcal{H}}$) instead of a single ${\mathcal{H}}$, we evaluated if the four ${\mathcal{H}}$ bring complementary information, either for each test case (by selecting different near-native poses) or for each test set (by performing well on different test cases).

#### Complementarity of the 4 ${\mathcal{H}}$ in a collection

**Fig. 7:**
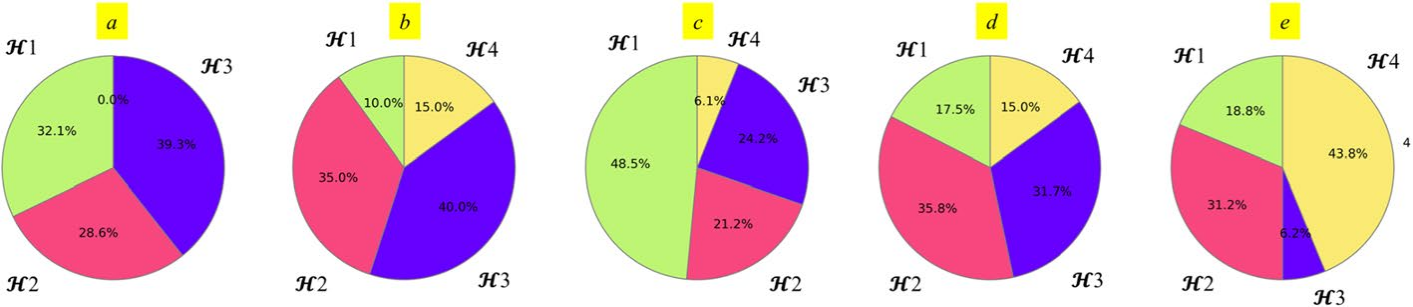
The percentage of cases within a test set, for which each of the 4 ${\mathcal{H}}$ in the collection is the best-performing one. **a** For collection 1 on test set 1. **b** For collection 2 on test set 2. **c** For collection 3 on test set 3. **d** For collection 4 on the united test set, suitable for validation of this collection’s performance. This set consists of the test cases belonging to all test sets, excluding sets 1, 2, 3 and 8. **e** For the collection 8 on test set 8 s

Out of 29 collections, the ones derived from the training sets 1, 2, 3, 4 and 8 are distinct (see Additional file 1: Table S3). The remaining collections are identical to the collection from training set 4. On the test set level, we can see that each single ${\mathcal{H}}$ is the best-performing (selects the highest number of near-natives) of the collection for 0% to 48% of the cases. This implies that there is no single ${\mathcal{H}}$ that universally outperforms others, encompassing half or more of the cases within a given test set (Fig. 7; Additional file 1: Table S4). Upon closer examination of individual test sets, we find that typically, two ${\mathcal{H}}$ options perform optimally for approximately 2/3 of the test cases within a set. Notably, in the largest test set, 4 (Fig. 7d), this relationship appears to be more balanced compared to the smaller test sets. This complies with the hypothesis that several different ${\mathcal{H}}$ are required to account for different binding modes, and that a few potentials better represent the diversity of RRM-ssRNA binding modes than one ${\mathcal{H}}$, by providing at least one well-suited ${\mathcal{H}}$ per case for most cases.

#### Best-performing ${\mathcal{H}}$ per case or per complex

For half of the cases, most of the near-natives in the TopC were selected by a single ${\mathcal{H}}$ out of four. If for each test case, we could use its best-performing ${\mathcal{H}}$ instead of the collection (and count near-natives in 20% top-ranked instead of pooling in the TopC), such modified application of HIPPO would reach a threefold enrichment for 77% cases (instead of 53% with the collection and 26% with ASF) and a fourfold enrichment for 62% cases (instead of 38% with the collection and 7% with ASF) (Fig. 6c; Additional file 1 Section 4, Table 4, Fig. S1). Furthermore, selecting only the 5% top-ranked poses would show a 12-fold enrichment for 39% cases (vs 4% cases with ASF). For the best-scored fragment per complex, a 12-fold enrichment was observed in 61% of complexes with HIPPO, while this is almost never achieved with ASF (7% of complexes). These numbers point toward the advantage of applying a single best-performing ${\mathcal{H}}$ per case rather than a collection, if one could predict which ${\mathcal{H}}$ to apply to which case. Such application appears to eliminate false positive poses, given by the less suitable potentials, thus providing a greater number of near-native poses among the top-ranked ones.

### Application of HIPPO and BP to new complexes

We searched the PDB and found six additional non-redundant RRM-ssRNA complexes solved between February 2021 and December 2023. This yielded another 29 protein-trinucleotide docking cases, referred to as "newRRM". We docked each case with ATTRACT and rescored the models with ASF and HIPPO, using the same parameters and without any re-training. The results are shown in Table 3. We found the ASF to perform considerably worse on the newRRM cases (14% success rate, vs 26% on the original benchmark). In contrast, HIPPO was successful in 16/29 (55%) of the new cases, a success rate very close to the original benchmark (53%). For all six complexes, there was at least one successful case. The average number of selected near-native models was also very close to the original benchmark (Table 4).

**Table 3:** Comparison of the ASF, HIPPO and BP success rates (%) over the cases and over the complexes

	‘newRRM’				‘nonRRM’			
	Per case		Best case per complex		Per case		Best case per complex	
	Over 60%	Over 80%	Over 60%	Over 80%	Over 60%	Over 80%	Over 60%	Over 80%
ASF	14	0	50	0	34	12	47	20
HIPPO	55	28	100	66	40	28	53	41
BP	90	70	100	100	72	54	85	69

The scoring is considered successful/very successful if the top20% contains at least 60%/80% of all sampled near-native poses

**Table 4:** Comparison of the average percentages of the near-natives in the top20% ranked poses by the ASF, HIPPO and BP

	‘newRRM’		‘nonRRM’	
	Per case	Best case per complex	Per case	Best case per complex
ASF	38	58	45	55
HIPPO	53	86	47	62
BP	83	99	73	85

Finally, to test HIPPO's robustness further, we also applied it to a benchmark of non-RRM complexes (519 cases), solved before August 2023 (Table 3 and 4, right columns). Here, HIPPO's success rate is much lower (40%), but still improves upon the ASF (34%). This improvement is statistically significant (p = 0.046, Fisher's exact test, two-tailed). Like for RRMs, the improvement upon ASF is more pronounced (28% vs 12%) when a stricter success criterion (> 80% in the top 20%) is applied.

### Comparison with other scoring functions

We compared ASF and HIPPO with 2 of the state-of-the-art RNA–protein scoring methods, namely DARS-RNP [36] and DRPScore [39] (chosen for their availability). We selected five random protein-trinucleotide cases where HIPPO worked well (> 75% near-natives selected in the top20% poses) and five random cases where HIPPO failed (< 25% near-natives selected in the top20% poses). Since HIPPO is successful in about half (53%) of the cases, and on average selects about half (55%) of the near-native poses in the top20%, we believe these ten cases to be representative of the overall performance of HIPPO.

For these ten cases, we evaluated DARS-RNP and DRPScore on the same ATTRACT models that had been scored by HIPPO. For each case, we first determined the distribution of scores by scoring 10,000 randomly selected poses. Then we scored all near-native poses, using the above distribution to determine their ranks.

We found DARS-RNP to be successful (> 60% of the near-natives in the top20%) for only two of the five cases where HIPPO worked well, and for none of the five cases where HIPPO failed. Likewise, DRPScore was successful for none of the ten cases. In general, we found that DARS-RNP, DRPScore and ASF select a similar percentage of near-natives (26–31% in the top20%), which is considerably below HIPPO.

### Comparison with other docking methods

We are not aware of any docking method other than ATTRACT that can systematically dock all conformers in our trinucleotide fragment library (2400–4800 conformers) against a protein in a reasonable amount of time. Therefore, we ran two docking methods, the 3dRPC web server [26] and AutoDock Vina [29, 30], with a highly favourable bias, namely by providing as input the "closest-to-bound" conformer, i.e. the conformer from the library that is closest in RMSD to the bound trinucleotide ssRNA fragment. For both docking methods, the docking of this single conformer took in the order of an hour. After docking, the ligand RMSD towards this bound reference was computed in the same way as for ATTRACT.

3dRPC and AutoDock Vina were run on 11 randomly selected docking cases. Among the 100 generated docking models (the server maximum), 3dRPC typically (median value) generated 1 near-native model, but with considerable variation: for four cases, a dozen or more near-natives were generated, while for four other cases, the 3dRPC server could not generate any near-natives at all, even though the closest-to-bound RNA conformer had been provided. Likewise, among 10,000 generated docking models, AutoDock Vina typically generated 29 (median value) near-native models, with > 100 near-natives for four cases and < 30 near-natives for six cases, of which two cases had no near-natives at all.

These results compare rather unfavourably to ATTRACT, which typically generated thousands of near-native structures (median value: 3145) and at least 100 near-natives for all but 12.5% of the cases, sampling in ~ 7 h ~ 10 000 poses for each of the 2400–4800 conformers in the library, not just the closest-to-bound one.

## Discussion

Despite the numerous biological roles of ssRNA-protein binding processes, there is still a lack of methods capable of addressing the dual challenges of the very high flexibility of ssRNA and the scarcity of its experimental structures. We previously developed a unique approach capable of modelling protein-bound ssRNA, by coarse-grained docking of ssRNA fragments with the ATTRACT docking software, followed by combinatorial assembly of geometrically compatible poses. Coarse-grained representation provides several advantages compared to all-atom representations. First, it accounts for inaccuracies in atomic positions coming either from bound/unbound conformational differences or experimental biases and resolution; second, it smoothes the energy landscape, which prevents the poses from getting stuck in shallow local minima; and third, it reduces the computation time. Our approach is successful in modelling the full ssRNA chain at high accuracy when conserved stacking contacts are known: the docking search space is reduced by constraints forcing the stacking of certain nucleotides on the conserved residues. In the absence of conserved contacts, this approach is limited by the poor sampling and low discriminatory power of the protein-RNA energy function of ATTRACT when applied to ssRNA fragments.

One reason is the main limitation of the fragment-based strategy, which stems from the concept of hotspot [13] and coldspot binding. A fragment by itself (taken in isolation) may have much stronger binding and hence lower real interaction energy in a region of the protein that is different from the binding region of that fragment when it is in the chain. This is a case of coldspot binding. The term “coldspot” refers to an area of the protein surface that can bind fragments relatively weakly. The opposite term, “hotspot”, refers to the part of the protein surface that binds fragments relatively strongly. Essentially, fragments that bind to the coldspots are only there because the adjacent fragments are tightly bound to the hotspots. From an energy perspective, binding to the coldspot leads to a shallow local energy minimum, whereas binding to the hotspot leads to a deeper (and possibly global) energy minimum. A mononucleotide tandem repeat sequence, such as the poly-U chain, provides a very intuitive example. For such an ssRNA, there are multiple overlapping native solutions for the same fragment sequence UUU that “compete” to be sampled and scored during the docking of UUU. As a consequence, there are usually one or two well-docked fragments, i.e. fragments with a lot of correctly ranked near-native poses, while the docking results for the remaining fragments are much worse. This limitation directly contributes to the so-called sampling and scoring problems.

When applying ssRNA’TTRACT, with typically a few thousand near-native poses sampled out of 10^7^ poses, the percentage of near-natives is less than 0.1%. In general, during assembly, low percentages of near-natives at the fragment level increase the probability of compatible non-native poses, leading to a prohibitive number of full-chain RNA models with an infinitesimally low percentage of quasi-native models. For direct applicability in the absence of conserved contacts, a very high enrichment is needed, followed by clustering and possibly refinement/rescoring with molecular dynamics, to arrive at an ensemble of perhaps a few hundred poses of which at least one is near-native.

In order to achieve such a high enrichment, we developed a new analytic approach for creating a scoring function for docking poses of coarse-grained ssRNA fragments, based on the frequencies of contact distances in near-native versus non-native poses. A specificity of our approach is to derive and combine a small set of potentials to better cover the diversity of ssRNA binding modes. We applied it to create HIPPO, a novel scoring function specifically for coarse-grained RRM-ssRNA fragment-based docking. On a benchmark of 57 RRM-ssRNA complexes. HIPPO demonstrates a better discriminatory power for near-native poses than the state-of-the-art ATTRACT scoring function (ASF).

The successfully and unsuccessfully scored cases are rather evenly distributed among the complexes. HIPPO's strengths and weaknesses are thus not likely to be attached to any specific type of complex, but rather to hot- and coldspots binding, meaning RNA fragments of a complex that are tightly and loosely attached to the protein respectively. This variability of docking performance over fragments is a difficulty inherent in a classical fragment-based docking approach, where each fragment must be docked (sampled and scored) within an accuracy threshold before the assembly. A way to tackle this is to ensure that at least one fragment per complex is very well docked and use each of its top-ranked poses as anchors to build a full RNA model by direct poses superposition followed by scoring. In the absence of evidence to identify the well-docked fragment from RNA sequence and protein structure, one would iteratively consider each fragment as such. We had previously applied a similar anchored docking of ssRNA on RRMs by using conserved stacking interactions between RRM aromatic residues and a nucleotide base as anchors [15]. Yet nearly half of RRM structures lack those conserved aromatics [21], and such a new hotspot approach would overcome this limitation. HIPPO will be better suited than ASF for this approach, since (i) more complexes have at least one successfully docked fragment compared to ASF, and (ii) the best-scored fragment in each complex has a higher enrichment for most complexes compared to ASF.

We found HIPPO to be rather robust in performance. Among an additional benchmark of recent RRM complexes, the success rate was essentially unchanged, even though ASF performed considerably worse. We also evaluated HIPPO on a benchmark of non-RRM complexes. Interestingly, HIPPO also shows improvement over ASF for these complexes, despite it being trained on RRMs only.

We have seen that the majority of the near-native poses in pooled top20% were often selected mostly by a single ${\mathcal{H}}$ of the four histogram sets and that for most cases (95%) the best-performing ${\mathcal{H}}$ of the collection (BP) performed better than the whole collection (Fig. 6c). A way to improve HIPPO’s performance would be to determine which ${\mathcal{H}}$ from the collection will perform the best on a given protein-fragment case. This would allow us to apply only this one ${\mathcal{H}}$ and avoid retaining false positives returned by the other three ${\mathcal{H}}$. Using the hypothetical knowledge of the BP yielded a 12-fold enrichment for nearly 40% of the test fragments—something which is achieved with ASF in only 4% of the cases. Most importantly, 61% of the complexes show such a 12-fold enrichment for at least one fragment. Under these conditions, the incremental modelling of entire ssRNA chains from best-docked fragments becomes viable, as top-ranked poses, selected by BP, could be clustered and resulting prototypes could be used as anchors. However, the problems of blindly identifying BP and selecting the best-docked fragments need to be solved first before this can become practical. This may be achieved with the help of supervised machine learning techniques based on the sequence of the fragment and the sequence or/and structure of the protein, and/or on the docking poses. Such a pre-trained classifier not only would drastically improve the performance of the scoring but could also give valuable insight into the most prevalent protein-ssRNA binding modes.

We see several tuning possibilities that might yield improved HIPPO performance. In particular, we will try to apply a stricter threshold for near-native poses, and see if, despite the increased sampling difficulties encountered, there would still be enough signal for HIPPO to succeed for high-accuracy poses. Moreover, as mentioned earlier, we face not only a scoring but also, primarily, a sampling problem in ssRNA docking. HIPPO can be considered as a pseudo-energy function, and as such, it is suitable for a sampling procedure based on energy minimisation that would not require derivability of the energy, such as a Monte Carlo approach [24]. We plan to test it against the current ATTRACT sampling procedure that uses ASF with gradient minimisation. Another possible way to apply HIPPO for the sampling is to convert each histogram into a differentiable function to be used directly in the ATTRACT gradient minimisation protocol. Finally, to further evaluate the generalisability of our approach for deriving scoring potentials, we plan to expand our benchmark from only RRM-ssRNA structures to a more general protein-ssRNA benchmark, as well as to our benchmark of protein-ssDNA structures [25].

### Supplementary Information

**Additional file 1. **Supplementary Materials.

## Data Availability

The source code of HIPPO is available via https://github.com/AnnaKravchenko/hippo. This repository contains the HIPPO scoring parameter set along with scripts to score protein-RNA docking models, together with an application guide. The data used for the study is available in the Supplementary Materials.
