# Supplementary material for: HIPPO: HIstogram-based Pseudo-POtential for scoring protein-ssRNA fragment-based docking poses
Source: BMC Bioinformatics. 2024 Mar 26;25:129. doi: 10.1186/s12859-024-05733-6 (PMC10964654; doi:10.1186/s12859-024-05733-6)
Supplement: Supplementary file 1 — Additional file 1. Supplementary Materials. [file 12859_2024_5733_MOESM1_ESM.docx]

Additional file 1

### **Table S1:** Benchmark of solved RRM-ssRNA structures

pdb_id: proteinChain1_firstAtom_lastAtom-proteinChain2_firstAtom_lastAtom -... rnaChain_firstAtom_lastAtom (numbers of the fragments used)

1A9N: A_1_162-B_1_94 Q_6_18 (2 3 4 5 6 7 11)

1B7F: A_1_167 P_3_12 (1 2 3 4 5 6 7 8)

1CVJ: A_1_169 M_2_9 (1 2 3 4 5 6)

1DRZ: A_1_91 B_48_57 (1 2 3 4 5 6 7)

1FJE: B_1_175 A_7_16 (1 2 3 4 5 7 8)

1FXL: A_1_167 B_1_8 (1 2 3 4 5 6)

1G2E: A_1_167 B_1_9 (1 2)

1M5K: C_1_92 B_35_44 (3 4 5 6)

1RKJ: A_1_175 B_8_15 (1 2 3 5)

1URN: A_1_96 P_6_12 (3 4 5)

1ZH5: A_1_181 D_2_9 (4 5 6)

2CJK: A_1_167 B_2_8 (1 2 3 4 5)

2G4B: A_1_172 B_3_7 (1 2 3)

2HYI: C_1_392-D_1_56-B_1_91-A_1_144 F_2_6 (1 2 3)

2J0S: A_1_391-C_1_143-D_1_89-T_1_44 E_1_6 (1)

2KG0: A_1_92 B_2_6 (1 2 3)

2KM8: B_1_84-C_1_167 A_1_13 (1 2 3 4 5 6 7 8 9 10)

2KXN: B_1_95 A_2_6 (1 2 3)

2M8D: B_1_91 A_2_8 (2 3 4 5)

2MGZ: A_1_94-B_1_105 C_2_12 (1 2 3 4 5 6 7 8 9)

2MKI: A_1_203 B_2_5 (1 2)

2MQO: A_1_105 B_2_6 (1 2 3)

2MQP: A_1_118 B_2_6 (1 2 3)

2MXY: A_1_105 B_2_7 (1 2 3 4)

2N3O: A_1_123 B_10_14 (1 2 3)

2RRA: A_1_99 B_2_6 (1 2 3)

2VOD: A_1_187 C_2_7 (4)

2VON: A_1_187 C_2_7 (3)

2XS7: A_1_86 B_2_4 (1)

3MOJ: B_1_75 A_46_49 (1 2)

3NNH: A_1_86-C_1_85 E_1_10 (2 3 4 5 6 7 8)

3RW6: A_1_245 H_11_15 (1 2 3)

4BS2: A_1_174 B_2_11 (1 2 3 4 5)

4CIO: A_1_97 B_2_7 (1 2 3 4)

4ED5: A_1_168 D_2_8 (1 2 3 4 5)

4F02: A_1_175-C_1_20 B_2_9 (6)

4N0T: A_1_363 B_11_29 (3 5 6 7 8 10 11 12 13 14 17)

4QQB: A_1_169-X_1_72 P_1_17 (1 2 3 4 5 6 7 8 10 11 12 13 14)

4YB1: P_1_92 R_8_11 (1)

5DET: A_1_91-B_1_94 Q_1_4 (1 2)

5HO4: A_1_179 B_2_5 (1 2)

5MPG: A_1_97 B_2_7 (1 2 3 4)

5MPL: A_1_102 B_2_6 (1 2 3)

5O1Y: A_1_163 B_2_4 (1)

5TF6: A_1_367 B_11_29 (11 12 13)

5WWE: A_1_174 B_2_5 (1 2)

5WWG: A_1_184 B_2_6 (1 2)

6ASO: A_1_369-B_1_95-C_1_79-D_1_59-E_1_79-F_1_75-G_1_67-H_1_83 I_8_26 (13 14)

6DCL: A_1_182-B_1_171 C_3_11 (1 2 3 4 5 6 7)

6F4G: A_1_175-B_1_95 C_9_19 (2 3 4 5 6)

6F4H: A_1_90 B_7_16 (5 6)

6G2K: A_1_80-B_1_80 R_1_6 (1 2 3 4)

6GBM: B_1_102 A_12_15 (1 2)

6GC5: A_1_79-B_1_76 E_1_5 (3)

6GD2: A_1_84-B_1_81 D_1_7 (2 3 4)

6GD3: B_1_83-C_1_84 P_1_6 (1 2 3 4)

6GX6: A_1_159 B_2_4 (1)

### **Table S2: Test sets**

Test set 1 (28 cases) : 1A9N 1DRZ 1M5K 1URN 4YB1 6F4G 6F4H

Test set 2 (20 cases) : 5HO4 5MPG 5MPL 5WWE 5WWG 6DCL

Test set 3 (33 cases): 1B7F 1FXL 1G2E 4ED5 4QQB

Test set 4 (12 cases): 6G2K 6GC5 6GD2 6GD3

Test set 5 (7 cases): 3NNH

Test set 6 (4 cases): 1ZH5 2VOD 2VON

Test set 7 (4 cases): 2HYI 2J0S

Test set 8 (16 cases): 4N0T 5TF6 6ASO

Test set 9 (6 cases): 2KXN 2RRA

Test set 10 (15 cases): 2CJK 2KM8

Test set 11 (13 cases): 2MGZ 4CIO

Test set 12 (7 cases): 1CVJ 4F02

Test set 13 (4 cases): 2MXY

Test set 14 (11 cases): 1FJE 1RKJ

Test set 15 (3 cases): 2N3O

Test set 16 (2 cases): 6GBM

Test set 17 (4 cases): 4BS2

Test set 18 (1 cases): 5O1Y

Test set 19 (2 cases): 2MKI

Test set 20 (1 cases): 6GX6

Test set 21 (3 cases): 2MQO

Test set 22 (3 cases): 2MQP

Test set 23 (4 cases): 2M8D

Test set 24 (1 cases): 2XS7

Test set 25 (3 cases): 2KG0

Test set 26 (2 cases): 5DET

Test set 27 (3 cases): 2G4B

Test set 28 (2 cases): 3MOJ

Test set 29 (3 cases): 3RW6

### **Table S3: Composition of the distinct HIPPO collections in terms of 𝓗**

| Collection id | 1 | 2 | 3 | 4 | 8 |
| --- | --- | --- | --- | --- | --- |
| Histogram sets 𝓗 | 2G4B-3-UUU  2KM8-1-UAU  4N0T-10-AGA  5MPG-1-UAG | 1M5K-4-GCA  3NNH-3-GUU  4N0T-10-AGA  6GD3-3-UUU | 1M5K-4-GCA  4N0T-10-AGA  6DCL-1-UAG  6DCL-5-UUA | 1M5K-4-GCA  4N0T-10-AGA  5MPG-1-UAG  6DCL-5-UUA | 1DRZ-3-UGC  2MQO-1-ACA  3NNH-3-GUU  5MPG-1-UAG |

### **Table S4:** Performance of ASF versus each unique 𝓗 on the test cases, where given 𝓗 is best-performing. Numbers in bold indicate better performance between ASF vs best-performing 𝓗.

| test case* where 𝓗 is best-performing | count of successfully scored cases | | count of best-scored cases | | avg % of selected near-natives per case | |
| --- | --- | --- | --- | --- | --- | --- |
|  | ASF | H | ASF | H | ASF | H |
| 2G4B-3-UUU | **1** | 0 | **3** | 1 | **51** | 23 |
| 2KM8-1-UAU | 0 | **7** | 1 | **9** | 32 | **67** |
| 4N0T-10-AGA | 15 | **47** | 6 | **55** | 39 | **75** |
| 5MPG-1-UAG | 18 | **47** | 5 | **50** | 50 | **82** |
| 1M5K-4-GCA | 12 | **26** | 3 | **33** | 48 | **79** |
| 3NNH-3-GUU | 0 | **5** | 3 | **7** | 27 | **54** |
| 6GD3-3-UUU | 2 | 2 | 1 | 1 | 82 | **87** |
| 6DCL-1-UAG | 4 | **6** | 1 | **6** | 63 | **82** |
| 6DCL-5-UUA | 3 | **23** | 0 | **25** | 31 | **92** |
| 1DRZ-3-UGC | 0 | 0 | **4** | 0 | **44** | 22 |
| 2MQO-1-ACA | 1 | **3** | 0 | **3** | 55 | **88** |
| superior performance count | 1 | 6 | 2 | 7 | 2 | 8 |

**Figure S1: Comparison of the percentage of selected near-natives by ASF vs the best-performing 𝓗**. Each pair of adjacent boxes shows the distribution of the results produced by each best-performing 𝓗 (purple) or ASF (pink) on the relative test cases for a range from 0% to 100% of all near-natives ranked in the 20% top-ranked poses.


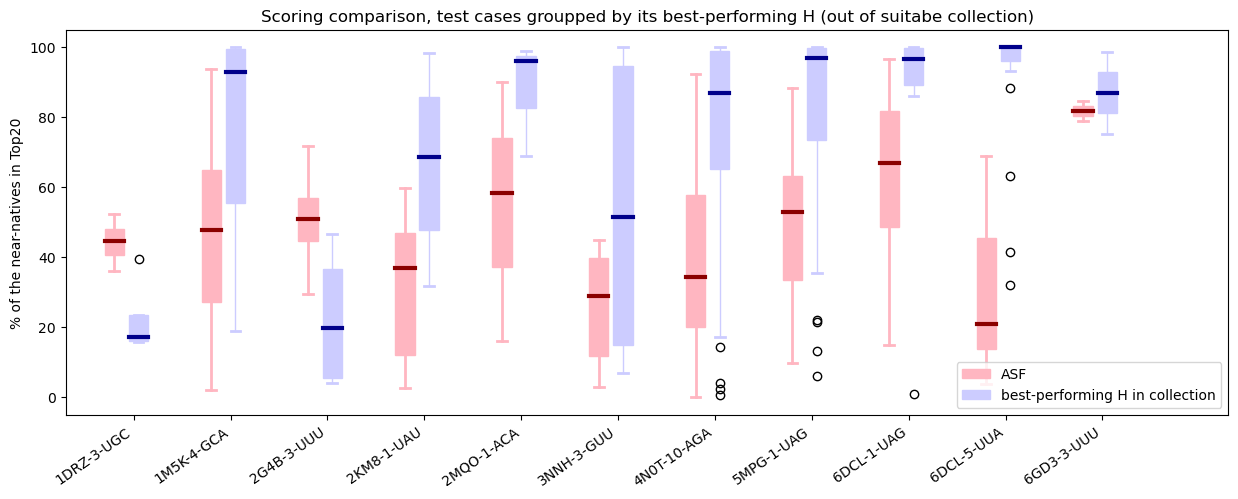


###

### **Table S5:** Benchmark of solved RRM-ssRNA structures ‘newRRM’

PDB_ID proteinChain_firstAtom-proteinChain_lastAtom rnaChain_firstAtom-rnaChain_lastAtom

| 6JVX  6YYM  7QDD  7VRL  7ZAP  7ZEW | A_29-A_115  A_580-A_726  B_114-B_201  B_107-B_208  A_141-B_237  A_1-A_114 | B_1-B_7  B_1-B_12  A_108-A_113  A_1-A_7  B_101-B_107  B_1-B_6 |
| --- | --- | --- |

### **Table S6:** Benchmark of solved protein-ssRNA structures (no RRM domains) ‘nonRRM’

PDB_ID proteinChain_firstAtom-proteinChain_lastAtom rnaChain_firstAtom-rnaChain_lastAtom

| 1ASY  1B23  1BMV  1DDL  1EC6  1ETF  1F7V  1HJI  1I9F  1J1U  1JBT  1JID  1K8W  1KQ2  1L9A  1LNG  1M8V  1M8X  1M8Y  1N1H  1NYB  1Q2R  1QFQ  1R3E  1RGO  1SI3  1SJ3  1TTT  1WMQ  1WNE  1WPU  1ZBH  1ZBN  1ZE2  1ZL3  2ANN  2ASB  2AZX  2BH2  2BQ5  2BU1  2C06  2CSX  2DB3  2DLC  2DRB  2FMT  2HGH  2I91  2IX1  2JLW  2JLX  2JPP  2KFY  2LI8  2MS1  2N8L  2N8M  2PLY  2PY9  2Q66  2QUX  2R7T  2R7V  2R7W  2R8S  2RSK  2RU7  2VNU  2XGJ  2XZL  2YJY  2ZZM  3AEV  3AMT | A_1-B_490  P_1-P_405  1_1-2_374  A_1-C_175  A_1-A_87  B_1-B_23  A_1-A_606  B_1-B_26  B_1-B_19  A_1-A_299  B_1-B_149  A_1-A_114  A_1-A_304  A_1-M_61  A_1-A_87  A_1-A_87  A_1-N_71  A_1-A_341  A_1-A_341  A_1-A1_264  A_1-A_22  A_1-A_376  B_1-B_35  A_1-A_305  A_1-A_70  A_1-A_117  P_1-P_95  A_1-A_405  A_1-B_143  A_1-A_476  A_1-B_147  A_1-D_289  B_1-B_17  A_1-A_300  A_1-A_302  A_1-A_148  A_1-A_226  A_1-A_377  A_1-A_418  A_1-C_129  A_1-C_129  A_1-B_110  A_1-A_464  A_1-A_420  X_1-X_339  A_1-A_437  A_1-A_314  A_1-A_87  A_1-A_520  A_1-A_643  A_1-A_450  A_1-A_451  A_1-B_53  A_1-A_102  A_1-A_63  A_1-A_55  A_1-A_191  A_1-A_191  A_1-B_198  A_1-B_66  A_1-A_519  A_1-B_121  A_1-A1_073  A_1-A1_073  A_1-A1_073  L_1-H_219  C_1-D_12  C_1-D_12  D_1-D_676  A_1-A_964  A_1-A_756  A_1-A_337  A_1-A_329  A_1-B_177  A_1-A_405 | S_72-S_75  R_71-R_74  M_1-M_5  D_1-D_7  D_5-D_16  A_23-A_27  B_13-B_22  A_6-A_9  A_23-A_27  B_35-B_38  D_14-D_17  B_13-B_16  B_10-B_14  R_2-R_7  B_36-B_39  B_24-B_27  S_1-S_6  C_2-C_8  C_1-C_10  B_1-B_4  B_10-B_14  E_11-E_14  A_6-A_10  C_7-C_11  D_2-D_9  B_2-B_9  R_49-R_58  D_73-D_76  C_1-C_7  B_2-B_6  C_2-C_7  E_7-E_10  A_14-A_17  C_10-C_14  B_9-B_15  B_8-B_16  B_2-B_11  C_32-C_37  C_2-C_12  R_6-R_12  R_5-R_11  C_2-C_5  C_33-C_39  E_2-E_5  Y_32-Y_35  B_32-B_35  C_73-C_77  B_5-B_8  D_8-D_14  B_1-B_13  C_2-C_6  C_2-C_7  C_8-C_13  B_2-B_6  B_2-B_7  B_6-B_9  B_2-B_7  B_1-B_7  C_11-C_14  E_6-E_12  X_2-X_5  C_11-C_16  X_2-X_7  X_2-X_5  X_2-X_7  R_66-R_78  A_2-A_12  A_2-A_12  B_2-B_9  C_2-C_5  B_1-B_8  C_1-C_10  B_35-B_41  C_2-C_11  B_33-B_41 | 3BOY  3BSB  3BSX  3BT7  3BX2  3BX3  3CUL  3DD2  3EX7  3FHT  3G9YA  3GIB  3HSB  3I5X  3IE1  3IEVA  3K49  3K5Q  3K5Y  3K5Z  3K61  3K62  3K64  3L26  3M7N  3M85  3MDG  3NVI  3O3I  3O8C  3OIJ  3PEW  3PF4A  3Q0M  3Q0Q  3Q0S  3QGB  3QGC  3QJJ  3QJL  3R2C  3R9W  3RC8  3RER  3T3O  3T5Q  3V71  484D  4ATO  4B8T  4BA2  4D25  4H5P  4I67  4J1G  4J7L  4JK0  4JNG  4JNXA  4JVYA  4K4U  4K4W  4KRE  4KRF  4Z0C  5YTS  5YTX  6KTC  6KUG  6RA4  6SQN  6UV1  6UV2  6UV4  6X5M | A_1-C_147  B_1-B_341  A_1-A_341  A_1-A_369  A_1-A_328  A_1-A_325  A_1-A_88  L_1-H_258  A_1-D_57  A_1-A_392  A_1-A_29  A_1-C_62  A_1-F_67  A_1-A_509  A_1-A_431  A_1-A_302  A_1-A_353  A_1-A_400  A_1-A_400  A_1-A_394  A_1-A_393  A_1-A_400  A_1-A_400  A_1-B_123  A_1-I_258  A_1-I_259  A_1-B_210  A_1-D_121  X_1-X_108  A_1-B_645  A_1-B_218  A_1-A_391  B_1-B_66  A_1-A_337  A_1-A_343  A_1-A_343  A_1-A_400  A_1-A_400  A_1-A_243  A_1-B_240  A_1-J_79  A_1-A_302  A_1-A_610  A_1-F_61  A_1-A_553  A_1-A_306  A_1-A_364  A_1-A_17  A_1-A_168  A_1-A_106  A_1-I_213  A_1-A_427  A_1-B_244  A_1-A_76  A_1-B_227  A_1-A_358  D_1-D_120  A_1-D_223  A_1-D_124  A_1-B_190  A_1-A_462  A_1-A_462  A_1-A_799  A_1-A_817  A_1-D_709  A_1-A_74  A_1-A_74  A_1-A_74  A_1-A_73  A_1-B_130  A_1-C_95  A_1-A_438  A_1-A_431  A_1-A_426  H_1-L_215 | D_2-D_21  C_2-C_9  C_2-C_10  C_6-C_14  C_2-C_9  C_2-C_8  C_29-C_38  B_13-B_16  F_2-F_6  C_1-C_6  C_2-C_6  H_1-H_9  X_2-X_7  B_3-B_10  E_1-E_4  D_1-D_9  B_2-B_10  B_1-B_9  B_2-B_9  B_2-B_9  B_2-B_9  B_2-B_9  B_2-B_9  C_4-C_8  Y_1-Y_5  X_1-X_4  C_2-C_5  E_9-E_24  A_11-A_14  C_1-C_6  C_5-C_10  B_1-B_5  R_2-R_5  C_2-C_8  B_2-B_8  B_2-B_8  B_2-B_9  B_2-B_9  Q_2-Q_12  X_2-X_12  R_2-R_8  B_24-B_34  E_1-E_5  K_2-K_8  B_1-B_5  C_1-C_8  B_1-B_7  B_8-B_18  G_2-G_5  B_1-B_5  R_1-R_4  D_1-D_6  E_2-E_14  B_1-B_4  E_2-E_44  B_1-B_5  B_1-B_5  L_1-L_42  B_8-B_15  D_1-D_6  B_1-B_5  B_1-B_6  R_1-R_9  R_1-R_12  C_1-C_13  B_1-B_4  B_1-B_4  V_1-V_4  B_1-B_4  M_2-M_9  Z_5-Z_12  C_2-C_7  C_2-C_7  C_1-C_7  R_15-R_19 |
| --- | --- | --- | --- | --- | --- |

## 
